# Efficacy and Safety of Long-Term and Short-Term Dual Antiplatelet Therapy: A Meta-Analysis of Comparison between Asians and Non-Asians

**DOI:** 10.3390/jcm9030652

**Published:** 2020-02-28

**Authors:** You-Jeong Ki, Jeehoon Kang, Jiesuck Park, Jung-Kyu Han, Han-Mo Yang, Kyung Woo Park, Hyun-Jae Kang, Bon-Kwon Koo, Hyo-Soo Kim

**Affiliations:** Department of Internal Medicine and Cardiovascular Center, Seoul National University Hospital, Seoul 03080, Korea; drkiyou@gmail.com (Y.-J.K.); medikang@gmail.com (J.K.); suckdood@naver.com (J.P.); hpcrates@gmail.com (J.-K.H.); hanname@hanmail.net (H.-M.Y.); nowkang@snu.ac.kr (H.-J.K.); hyosoo@snu.ac.kr (H.-S.K.)

**Keywords:** dual anti-platelet therapy, percutaneous coronary intervention, meta-analysis

## Abstract

While dual antiplatelet therapy (DAPT) is essential after percutaneous coronary intervention (PCI), the optimal duration is affected by various factors. However, the effect of ethnicity on DAPT duration has not been fully evaluated. In this study, we evaluated the different effect of DAPT duration by ethnicity. We searched Pubmed, Embase, Cochrane library, and relevant websites to search for randomized clinical trials (RCT) assessing the clinical impact of long term DAPT (L-DAPT) and short term DAPT (S-DAPT). Studies were divided by ethnicity, and we compared the efficacy and safety of DAPT duration in each ethnic group. Thirteen RCTs including 38,255 patients (five East Asian studies and eight non–East Asian studies) were eligible for analysis. For the primary outcome, L-DAPT showed a significantly lower rate of primary outcome only in non–East Asians (S-DAPT vs. L-DAPT, odds ratio (OR) = 1.16, 95% confidence interval (CI): 1.02–1.32, *p* = 0.02), while in East Asians, the effect of S-DAPT and L-DAPT were comparable. S-DAPT significantly increased ischemic events only in non–East Asians (S-DAPT vs. L-DAPT, OR = 1.24, 95% CI: 1.09–1.42, *p* <0.01), while bleeding events were decreased by S-DAPT in both ethnicities. These results demonstrate that the adequate DAPT duration after PCI may be different in East Asians.

## 1. Introduction

Dual antiplatelet therapy (DAPT) with aspirin and a P2Y12 inhibitor is recommended after percutaneous coronary intervention (PCI) to reduce stent thrombosis (ST) and ischemic events [1]. However, the ideal duration of DAPT after stenting is still debatable. Longer usage of DAPT reduces the risk of ischemic events but increases the risk of bleeding events. Thus, there is a fundamental trade-off between ischemic risk and bleeding risk that needs to be considered while deciding the duration of DAPT [2]. Meanwhile, owing to the advances in current devices, including new generation stents, the minimum DAPT duration required to avoid stent-associated ischemic outcomes is decreasing.

Recently, the Academic Research Consortium (ARC) has published a white paper to define high bleeding risk (HBR) stressing the careful usage of DAPT in patients undergoing PCI [3]. Because more and more candidates for PCI are at high risk of bleeding, it is not surprising that various clinical trials, including the LEADERS FREE trial and the ONYX ONE trial, are being conducted to confirm the safety of a shorter duration of DAPT in patients with HBR [4,5].

Ethnicity, in particular the East Asian ethnicity, may be a factor that contributes to higher bleeding risk. We, among others, have reported that East Asians may have higher rates of bleeding but have comparable or lower rates of ischemic events than Caucasian patients [6,7,8,9]. However, there is a paucity of studies about the effect of ethnicity of the ischemia/bleeding risk.

Therefore, in the current study, we performed a meta-analysis to compare the differences in the efficacy and safety of a short course of DAPT among East Asians and non–East Asians to add to the evidence on the relationship between ethnicity and duration of DAPT.

## 2. Materials and Methods

### 2.1. Data Sources and Study Strategy

We searched Pubmed, Embase, Cochrane library, and relevant websites to search for randomized clinical trials (RCT) assessing the clinical impact of long term DAPT (L-DAPT) and short-term DAPT (S-DAPT). The following search terms were used ((“aspirin” AND “P2Y12 receptor inhibitor” or “clopidogrel” or “Plavix” or “prasugrel” or “Effient” or “ticagrelor” or “Brilinta” or “thienopyridine”) or “DAPT” or “dual antiplatelet therapy”) AND (“drug eluting stent” or “DES”). Search strategies included both the Medical Subject Heading term and text word searches. To ensure a comprehensive search of the literature, we also manually searched the reference lists of the included studies, recent reviews, editorials, and meta-analyses. No language restrictions were applied.

### 2.2. Study Selection, Data Extraction and Quality Assessment

All the RCTs comparing different duration of DAPT after PCI were included in the meta-analysis. Two investigators independently reviewed the studies and extracted the results in a formalized dataset. Discrepancies were resolved by consensus by group discussion. The quality of the eligible RCTs was assessed using the Cochrane Collaboration’s tool for assessing the risk of bias for RCTs. The search period took place form October 2008 to October 2018.

### 2.3. Outcomes and Definitions

Study groups were classified as S-DAPT and L-DAPT. S-DAPT was defined as the per protocol shorter duration of DAPT after stent insertion. L-DAPT refers to the group using DAPT longer duration in each study. The primary endpoint was a composite of death, cardiac death, myocardial infarction (MI), stroke, revascularization or bleeding events, but the exact definition was different for each study. The ischemic outcome was defined as composite of cardiac death, MI, ST, stroke or revascularization. Additional outcomes included each components of primary outcome or ischemic outcome, and bleeding event. The exact definition of the primary, ischemic, and bleeding outcomes of each study was described in Table 1.

### 2.4. Statistical Analysis

A random-effects model and fixed effect model with inverse variance weighting were used to calculate odds ratio (OR) and 95% confidence interval (CI), associated with S-DAPT versus L-DAPT for above end point. Forest plot was used to calculate the overall effect of studies. Heterogeneity between studies was assessed using Cochrane’s Q test and I^2^ statistic, which denotes the percentage of total variation across study that is a result of heterogeneity rather than chance. I^2^ represents an estimate of the degree of inconsistency among studies, with scores of 25%, 50%, and 75% representing, respectively, low, moderate, or high inconsistency. Heterogeneity considered significant if the *p* value was less than 0.05. Funnel plots, Begg’s test, and Egger’s test were used to assess the publication bias. Analyses were performed with R programming language, version 3.5.0 (R Foundation for Statistical Computing, Vienne, Austria). The PRISMA statement for reporting systematic reviews and meta-analyses of studies was used for the method of the present study.

## 3. Results

### 3.1. Study Outlines and Characteristics

**Figure 1:**
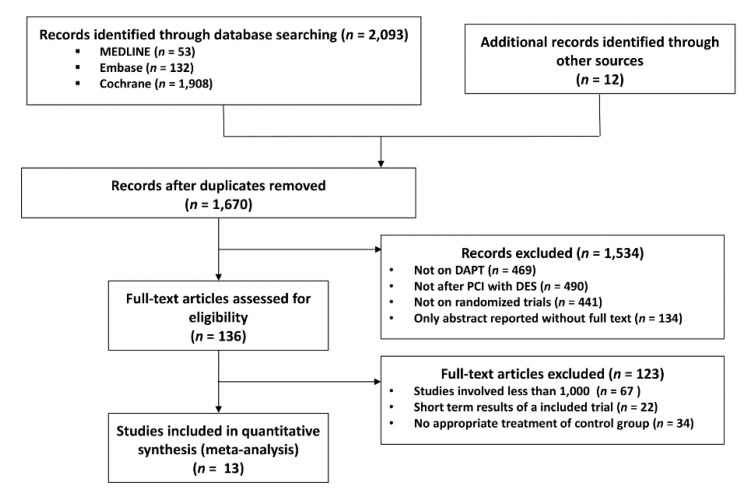
Meta-analysis flow diagram. DAPT, dual anti platelet therapy; PCI, percutaneous coronary intervention; DES, drug eluting stent.

We initially identified 1670 publications by electronic search and on initial screening. After careful assessment of titles and abstracts, 136 studies were identified for review of full-text manuscripts. By full-text review, 123 studies were excluded mainly because of the non-randomized study design and short follow-up period. Finally, 13 studies were selected for our meta-analysis (Figure 1). Among these, the EXCELLENT [10], RESET [11], DES-LATE [12], IVUS-XPL [13], and NIPPON [14] studies were conducted in East Asian countries (13,312 patients), while the PRODIGY [15], OPTIMIZE [16], ARCTIC INT [17], DAPT [18], SECURITY [19], ISAR-SAFE [20], ITALIC [21], and OPTIDUAL [22] were trials performed in non–East Asian countries (24,943 patients). The baseline characteristics of the study are listed in Table 2. Among 38,255 patients, 19,054 and 19,201 patients were randomized to S-DAPT and L-DAPT groups, respectively. Descriptors of the included studies and baseline characteristics of the patients enrolled in these studies are provided in Table 2.

### 3.2. Clinical Outcomes

**Figure 2:**
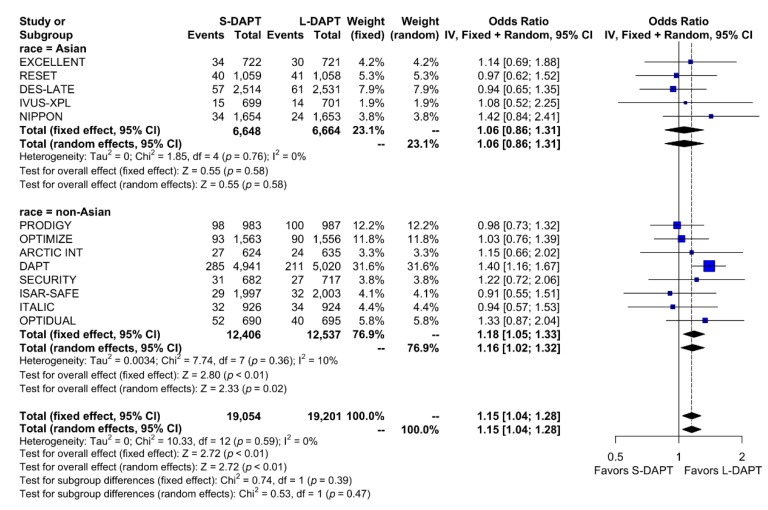
Primary outcome in randomized clinical trials. S-DAPT, short duration dual antiplatelet therapy; L-DAPT, long duration dual antiplatelet therapy; CI = confidence interval.

The rate of the primary analysis outcome was 4.1% (1555 events in 38,255 patients) in the total population. When stratified according to ethnicity and DAPT duration, the rate of the primary analysis outcome was similar between S-DAPT and L-DAPT (event rate 2.7% (180 of 6648 patients) vs. 2.6% (170 of 6664 patients)) among East Asians. Among non–East Asians, the event rate was 5.2% (647 of 12,406 patients) in the S-DAPT group, which was higher than that in the L-DAPT group (4.5%, 558 of 12,537 patients). The risk of occurrence of the primary outcome significantly increased in the S-DAPT compared with that in the L-DAPT among non–East Asians (S-DAPT vs. L-DAPT, OR = 1.16, 95% CI: 1.02–1.32, *p* = 0.02), but not among East Asians (S-DAPT vs. L-DAPT, OR = 1.06, 95% CI: 0.86–1.31, *p* = 0.58) (Figure 2). The funnel plot, Begg’s test, and Egger’s test did not reveal any publication bias (Begg’s test *p* = 0.4354, Egger’s test *p* = 0.1843) (Supplementary Materials, Figure S1).

**Figure 3:**
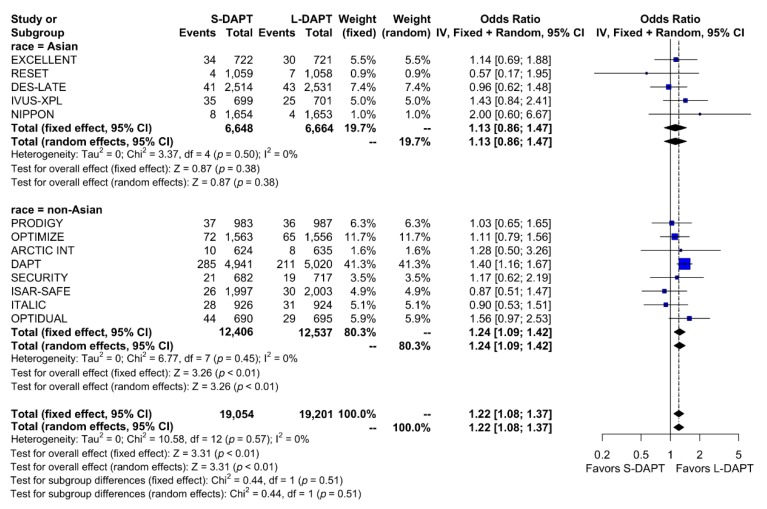
Ischemic outcomes in randomized clinical trials. S-DAPT, short duration dual antiplatelet therapy; L-DAPT, long duration dual antiplatelet therapy; CI, confidence interval.

We analyzed the effect of DAPT on ischemic outcomes defined as a composite of cardiac death, MI, ST, stroke or revascularization according to ethnicity. Among non–East Asians, ischemic events occurred in 523 of 12,406 patients (4.2%) treated with S-DAPT and 429 of 12,537 patients (3.4%) treated with L-DAPT. Among East Asians, ischemic events occurred in 122 of 6,648 patients (1.8%) treated with S-DAPT and in 109 of 6,664 patients (1.6%) treated with L-DAPT. S-DAPT was associated with higher rates of ischemic events than L-DAPT among non–East Asians (S-DAPT vs. L-DAPT, OR = 1.24, 95% CI: 1.09–1.42, *p* < 0.01), whereas L-DAPT was comparable to S-DAPT among East Asians (S-DAPT vs. L-DAPT, OR = 1.13, 95% CI: 0.86–1.47, p = 0.38; p for interaction 0.51) (Figure 3).

**Figure 4:**
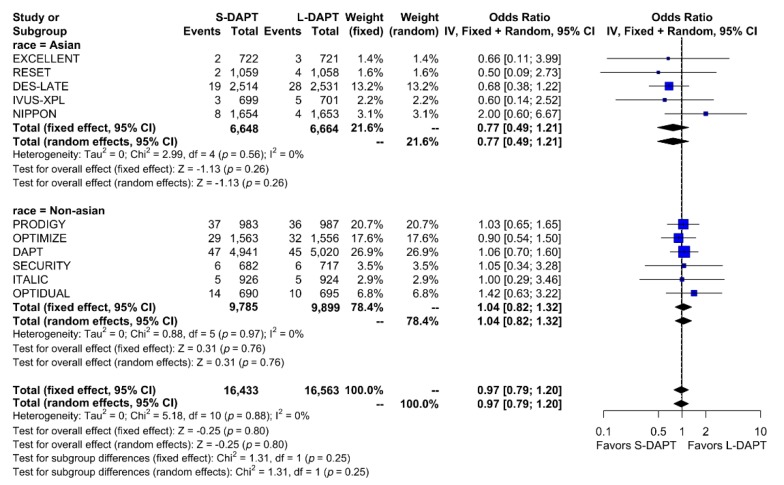
Cardiac death in randomized clinical trials. S-DAPT, short duration dual antiplatelet therapy; L-DAPT, long duration dual antiplatelet therapy; CI, confidence interval.

**Figure 5:**
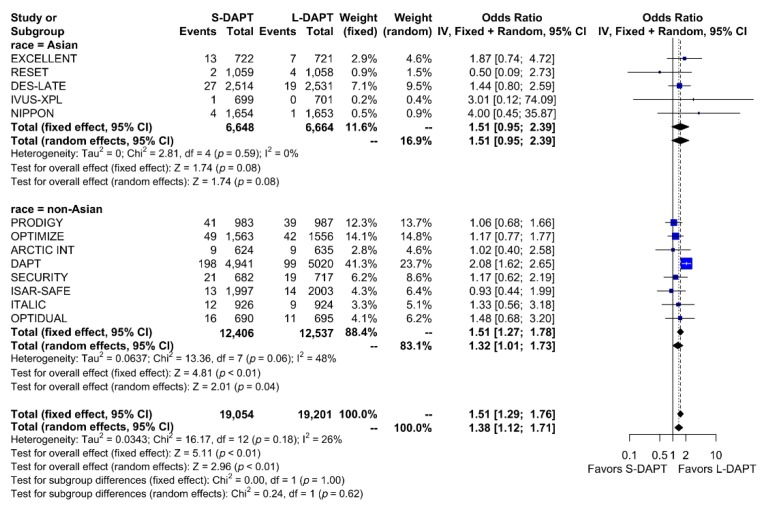
Myocardial infarction in randomized clinical trials. S-DAPT, short duration dual antiplatelet therapy; L-DAPT, long duration dual antiplatelet therapy; CI, confidence interval.

**Figure 6:**
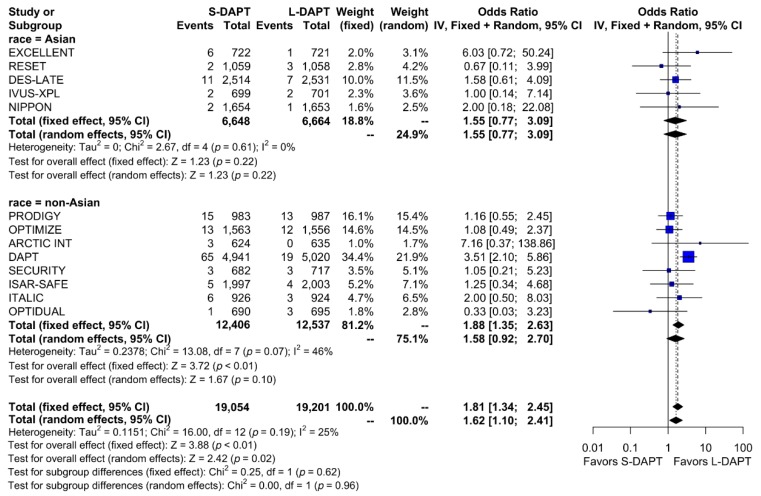
Stent thrombosis in randomized clinical trials. S-DAPT, short duration dual antiplatelet therapy; L-DAPT, long duration dual antiplatelet therapy; CI, confidence interval.

Other secondary endpoints, such as cardiac death was evaluated in 11 studies. No statistical difference in cardiac mortality was noted between the S-DAPT and L-DAPT groups among East Asians and non–East Asians. (S-DAPT vs. L-DAPT, OR = 0.77, 95% CI: 0.49–1.21, *p* = 0.26 in East Asians, OR = 1.04, 95% CI: 0.82–1.32, *p* = 0.76 in non–East Asians) (Figure 4). For MI, which was evaluated in 13 studies, L-DAPT was associated with lower MI rates than S-DAPT among both East Asians and non–East Asians; however it was statistically significant among non–East Asians. (S-DAPT vs. L-DAPT, OR = 1.51, 95% CI: 0.95–2.39, *p* = 0.08 in East Asians, OR = 1.32, 95% CI: 1.01–1.73, *p* = 0.04 in non–East Asians). No significant interaction was noted between East Asians and non–East Asians (*p* for interaction 0.62) (Figure 5). L-DAPT was associated with a lower risk of ST, which was slightly more pronounced in non–East Asians than in East Asians. (S-DAPT vs. L-DAPT, OR = 1.55, 95% CI: 0.77–3.09, *p* = 0.22 in East Asians, OR = 1.58, 95% CI: 0.92–2.70, *p* = 0.10 in non–East Asians; *p* for interaction 0.96) (Figure 6).

**Figure 7:**
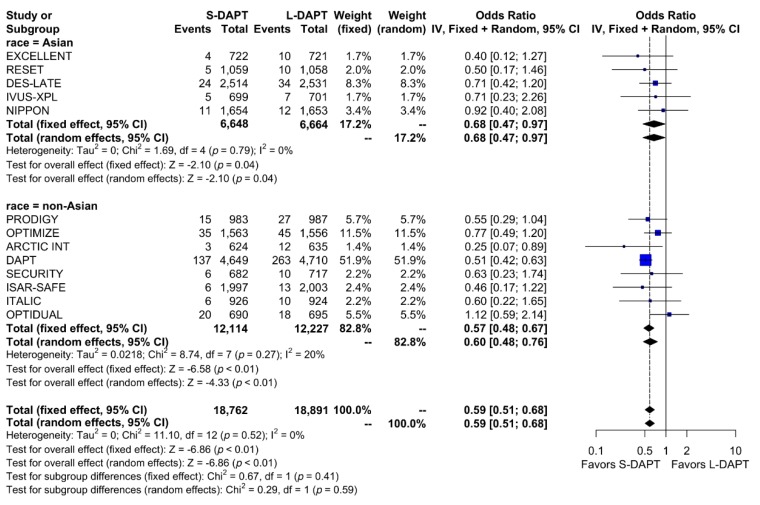
Bleeding in randomized clinical trials. S-DAPT, short duration dual antiplatelet therapy; L-DAPT, long duration dual antiplatelet therapy; CI, confidence interval.

A total of 748 bleeding events (2.0%) occurred among the total population. When stratified based on the ethnicity and DAPT duration, bleeding events occurred in 0.7% (49 out of 6,648 patients) of patients treated with S-DAPT and in 1.1% (73 out of 6,664 patients) of patients treated with L-DAPT among the East Asian population. Among the non–East Asians, bleeding events occurred in 1.9% (228 out of 12,114 patients) of patients treated with S-DAPT and in 3.3% (398 of 12,227 patients) of patients treated with L-DAPT. S-DAPT significantly reduced the risk of bleeding events among both East Asians and non–East Asians (S-DAPT vs. L-DAPT, OR = 0.68, 95% CI: 0.47–0.97, *p* = 0.04 in East Asians, OR = 0.60, 95% CI: 0.48–0.76, *p* < 0.01 in non–East Asians; *p* for interaction 0.59) (Figure 7).

## 4. Discussion

The current meta-analysis is the first study to compare the duration of DAPT from the racial standpoint. The main findings of this study are as follows: (1) L-DAPT showed a significantly lower rate of primary outcome among non–East Asians, whereas the effects of S-DAPT and L-DAPT were comparable among East Asians. (2) L-DAPT could significantly decrease ischemic outcomes among non–East Asians. (3) L-DAPT was associated with a lower risk of ST, which was slightly more prominent in non–East Asians than in East Asians. (4) S-DAPT decreased the risk of bleeding events in both ethnicities, compared to L-DAPT. Our meta-analysis intended to compare the bleeding/ischemic risk between East Asians and non–East Asians, suggesting that differences in ischemic/bleeding tendencies over the duration of DAPT are affected by ethnicity.

### 4.1. Ideal Duration of DAPT after PCI

Although there is no disagreement with using DAPT after PCI, the ideal duration of DAPT after stenting is debatable. Long-term use of DAPT is helpful for the prevention of late ST and ischemic events [1,23], whereas this is associated with a higher bleeding risk. Thus, there is trade-off for deciding the duration of DAPT [2].

The recommended minimum duration of DAPT has shortened with the emergence of several studies demonstrating the safety of short duration of DAPT. In the meta-analysis by Palmerini et al., three-month or six-month DAPT showed a similar rate of major adverse cardiovascular events and lower rate of bleeding compared with one-year DAPT [24]. Similar results were observed in another meta-analysis [25]. On the basis of these trials, the current guidelines recommend a varied and shorter DAPT duration [26,27].

Currently it has been established that one size does not fit all for the usage of DAPT. Because many factors were proposed to influence the ischemic/bleeding tradeoff, the use of bleeding/ischemia scoring system (The DAPT score and PRECISE-DAPT score) to evaluate the benefits and risks of different DAPT durations recommended in the current guidelines [27]. According to ARC HBR definition, patients are considered as HBR if at least one major or two minor criteria are met. Factors associated with an increased bleeding risk after PCI includes old age (≥75 years), renal disease, liver disease, active cancer, anemia, low platelet count, previous intracranial hemorrhage, stroke, brain arteriovenous malformation, bleeding diathesis, prior bleeding or transfusion history, long-term use of oral anticoagulation, non-steroidal anti-inflammatory drugs, or steroids, and recent trauma or surgery (3).

### 4.2. Effect of Ethnicity on DAPT Duration

Among various factors that were suggested to affect the ischemic/bleeding tradeoff, the effect of ethnicity has not been fully evaluated. In particular, East Asians have been suggested to have higher bleeding risk and lower ischemic risk. There are some possible explanations of this trend.

First, East Asians have lower body mass index (BMI) than non–East Asians. Obesity is associated with a pro-thrombotic status. Overweight patients had higher levels of annexin V-micro particles, endothelial-derived, leukocyte-derived, and tissue factor-bearing micro particles than controls [28].

Second, the differences in genetic polymorphism among the races may be related with differences in thrombogenicity. In the MESA (Multi-Ethnic Study of Atherosclerosis) study, differences are seen in the hemostatic factors and plasma endothelial marker levels [29]. African-Americans generally had the most thrombogenic and dysfunctional endothelial profile (fibrinogen, factor VIII, D-dimer, plasmin-anti plasmin, and von Willebrand factor), followed by Hispanics and Caucasians, and then East Asians. East Asians had the highest levels of plasminogen activator inhibitors-1 than other races.

Thirdly, there are associations between inflammation and atherothrombosis. In the SWAN (Study of Women’s Health across the Nation) study, the highest C-reactive protein (CRP) levels were found among African-American women, followed by Hispanics, Caucasians, and East Asians [30].

Regarding clopidogrel, platelet inhibition is mediated by cytochrome P-450 isoenzymes (CYP) [31]. Various mutations of CYP are related to a decrease in platelet responsiveness to clopidogrel, while many studies have shown a relationship between clopidogrel non-responsiveness and adverse clinical ischemic events [32,33]. Asians are known to have a higher rate of CYP gene polymorphisms leading to a higher rate of clopidogrel non-responsiveness. Despite the previous results of clopidogrel non-responsiveness, moderate bleeding occurred more frequently among Asians than that among Caucasians, and ethnicity was an independent predictor of the moderate bleeding complications [34]. This unique phenomenon among Asians was defined as the “East-Asian paradox,” leading to a hypothesis that a different therapeutic window for platelet inhibition should be applied to Asians.

Moreover, following the improvement in coronary stents, the optimal duration of DAPT is reducing to decrease the bleeding risk while maintaining ischemic event prevention [2]. In addition to the LEADERS FREE study, the ONYX ONE study also showed that Resolute Onyx DES was safe and effective for HBR patients with 1-month DAPT [4,5]. Considering the current trend of reducing DAPT duration and differences in bleeding tendency according to ethnicity, the DAPT period may be reduced in East Asians.

### 4.3. Limitations

There are several limitations to this meta-analysis. First, the ethnicity was determined based on the sites of the participating studies. Although Caucasians are very rarely included in the RCTs on East Asians, we cannot exclude a small possibility of the ethnic mix-up in non-Asian studies. However, RCTs on both East Asians and non–East Asians are affected. Second, the difference effects of duration of DAPT seen may related to the difference of the East Asian and non–East Asian studies, rather than the racial differences. Therefore, caution is needed in interpreting the overall effect as the sole effect of racial difference. Third, each study used a different definition for S-DAPT and L-DAPT. However, because our study was planned to compare the relative effect of S-DAPT and L-DAPT and not to determine an optimal DAPT duration, we assume that this heterogeneity would not largely influence the results of our study. Fourth, most of these studies were underpowered to detect differences in outcomes because of the different study designs and lower event rates; while the primary endpoint definitions were heterogeneous between studies, we additionally analyzed the ischemic outcomes and bleeding outcomes, respectively to overcome this limitation.

## 5. Conclusions

Our data suggest that the ischemia/bleeding tradeoff and thus the therapeutic range of DAPT may be different between East Asians and non–East Asians. In the present analysis of RCTs, L-DAPT compared to S-DAPT showed significantly lower rate of ischemic outcomes only in non–East Asian, while bleeding events were decreased by S-DAPT in both ethnicities. These results suggest that the optimal duration of DAPT in East Asians could be shorter than that compared to non–East Asians.

## Figures and Tables

**Table 1:** Current large-scale randomized clinical trials (RCTs) comparing the efficacy and safety of long-term and short-term dual antiplatelet therapy (DAPT).

Study	Study Performed Nation	Year	DAPT Duration and Patient Number	Trial Design	Primary Endpoint	Ischemic Outcome	Bleeding Outcomes	Result
EXCELLENT	East Asia (Korea)	2012	6 months (*n* = 722) 12 months (*n* = 721)	Non-inferiority	Cardiac death/MI /Ischemia driven TVR	Cardiac death/MI /Ischemia driven TVR	Any bleeding	Non-inferiority of 6 months DAPT demonstrated
PRODIGY	Europe	2012	6 months (*n* = 751) 24 months (*n* = 750)	Superiority	Death/MI/Stroke	Cardiac death	TIMI major or minor	Superiority of 24 months DAPT was not demonstrated
RESET	East Asia (Korea)	2012	3 months (*n* = 1059) 12 months (*n* = 1058)	Non-inferiority	Cardiac death/MI/ST /TVR/Major bleeding	Cardiac death/MI	TIMI major or minor	Non-inferiority of 3 months DAPT demonstrated
OPTIMIZE	South America	2013	3 months (*n* = 1563) 12 months (*n* = 1556)	Non-inferiority	Death/MI/Stroke/ Major bleeding	Cardiac death/MI/ST	Any bleeding	Non-inferiority of 3 months DAPT demonstrated
ARCTIC INT	Europe	2014	12 months (*n* = 624) 24 months (*n* = 635)	Superiority	Death/MI/ST/Stroke/ urgent revascularization	ST/urgent revascularization	STEEPLE major or minor	Superiority of 24 months DAPT was not demonstrated
DAPT	America, Australia, Europe	2014	12 months (*n* = 4941) 30 months (*n* = 5020)	Superiority	ST and All cause death/MI/Stroke	Death/MI/Stroke	BARC 2, 3, or 5	Superiority of 30 months DAPT was demonstrated
DES-LATE	East Asia (Korea)	2014	12 months (*n* = 2514) 36 months (*n* = 2531)	Equivalence	Cardiac death/MI /Stroke	Cardiac death/MI	TIMI major bleeding	Equal effect of 12 months and 24 months DAPT
SECURITY	Europe	2014	6 months (*n* = 682) 12 months (*n* = 717)	Non-inferiority	Cardiac death/MI/ST/Stroke /Major Bleeding	MI	BARC 2, 3, or 5	Non-inferiority of 6 months DAPT demonstrated
ISAR-SAFE	Europe	2015	6 months (*n* = 1997) 12 months (*n* = 2003)	Non-inferiority	Death/MI/ST/Stroke /Major bleeding	Death/MI/ST/Stroke	TIMI major or minor	Non-inferiority of 6 months DAPT demonstrated (premature termination)
ITALIC	Europe, Middle East	2015	6 months (*n* = 926) 24 months (*n* = 924)	Non-inferiority	Death/MI/TVR Stroke/Major bleeding	Death/MI/Stroke	TIMI major or minor	Non-inferiority of 6 months DAPT demonstrated
IVUS-XPL	East Asia (Korea)	2016	6 months (*n* = 699) 12 months (*n* = 701)	Equivalence	Cardiac death/MI/ Stroke/Major bleeding	Cardiac death/MI/Repeat revascularization	TIMI major bleeding	Equal effect of 6 months and 12 months DAPT
OPTIDUAL	Europe	2016	12 months (*n* = 690) 48 months (*n* = 695)	Superiority	Death/MI/Stroke/ Major bleeding	Death/MI/Stroke	TIMI major or minor	Superiority of 48 months DAPT was not demonstrated
NIPPON	East Asia (Japan)	2017	6 months (*n* = 1886) 18 months (*n* = 1887)	Non-inferiority	Death/MI/Stroke/ Major bleeding	Cardiac death	BARC 3 or 5 bleeding	Non-inferiority of 6 months DAPT demonstrated

RCT, randomized controlled trials; DAPT, dual antiplatelet therapy; EXCELLENT, efficacy of Xience/Promus versus cypher to reduce late loss after stenting; MI, myocardial infarction; TVR, target vessel revascularization; PRODIGY, prolonging dual antiplatelet treatment after grading stent-induced intimal hyperplasia study; TIMI, thrombolysis in myocardial infarction; RESET, real safety and efficacy of three-month dual antiplatelet therapy following Endeavor zotarolimus-eluting stent implantation; ST, stent thrombosis; OPTIMIZE, optimized duration of clopidogrel therapy following treatment with the zotarolimus-eluting stent in real-world clinical practice; ARCTIC INT, dual-antiplatelet treatment beyond one year after drug-eluting stent implantation; STEEPLE, safety and efficacy of enoxaparin in percutaneous coronary intervention patients; DES-LATE, duration of clopidogrel therapy after drug-eluting stent; SECURITY, second generation drug-eluting stent implantation followed by six- versus 12-month antiplatelet therapy; ISAR-SAFE, safety and efficacy of six months dual antiplatelet therapy after drug-eluting stenting; ITALIC, is there a life for DES after discontinuation of clopidogrel; IVUS-XPL, impact of intravascular ultrasound guidance on outcomes of XIENCE PRIME stents in long lesions; OPTIDUAL, optimal dual antiplatelet therapy; NIPPON, Nobori dual antiplatelet therapy as appropriate duration.

**Table 2:** Baseline patient characteristics in the including studies.

Study	N	Follow-up (Months)	Age (Years)	Male (%)	DM (%)	ACS (%)	Clopidogrel/Prasugrel/Ticagrelor	First Generation DES/Second Generation DES (%)	Prior MI (%)	Timing of Randomization
EXCELLENT	1443	12	63	931 (65%)	550 (38%)	744 (52%)	100/0/0	25/75	74 (5%)	Index PCI
PRODIGY	1970	24	68	1511 (77%)	477 (24%)	1465 (74%)	100/0/0	25/50	528 (27%)	1 month
RESET	2117	12	62	1347 (64%)	621 (29%)	1156 (55%)	100/0/0	NA	36 (2%)	Index PCI
OPTIMIZE	3119	12	62	1974 (63%)	1103 (35%)	996 (32%)	100/0/0	0/100	1083 (35%)	Index PCI
ARCTIC INT	1259	17	64	1011 (80%)	420 (33%)	NA	91.4/8.6/0	42/63	383 (30%)	12 months
DAPT	9961	33	62	7435 (75%)	3037 (30%)	4251 (43%)	65/35/0	38/60	2118 (22%)	12 months
DES-LATE	5045	36	62	3498 (69%)	1418 (28%)	3063 (61%)	100/0/0	64/36	195 (4%)	12 months
SECURITY	1399	12	65	1080 (77%)	429 (31%)	442 (32%)	99.4/0.2/0.4	0/100	289 (21%)	Index PCI
ISAR-SAFE	4000	15	67	3223 (81%)	979 (24%)	1601 (40%)	100/0/0	27/72	1007 (25%)	6 months
ITALIC	1850	24	62	1483 (80%)	685 (37%)	806 (44%)	98.2/0.1/1.7	0/100	282 (15%)	6 months
IVUS-XPL	1400	12	64	964 (69%)	506 (36%)	686 (49%)	100/0/0	0/100	63 (5%)	Index PCI
OPTIDUAL	1385	34	64	1115 (81%)	435 (31%)	501 (36%)	100/0/0	34/66	241 (17%)	12 months
NIPPON	3307	18	67	2616 (79%)	1254 (38%)	1079 (33%)	97/0.1/0	0/100	396 (12%)	Index PCI

DM, diabetes mellitus; ACS, acute coronary syndrome; DES, drug eluting stent; MI, myocardial infarction; EXCELLENT, efficacy of Xience/Promus versus cypher to reduce late loss after stenting; PCI, percutaneous coronary intervention; PRODIGY, prolonging dual antiplatelet treatment after grading stent-induced intimal hyperplasia study; RESET, real safety and efficacy of three-month dual antiplatelet therapy following endeavor zotarolimus-eluting stent implantation; OPTIMIZE, optimized duration of clopidogrel therapy following treatment with the Zotarolimus-eluting stent in real-world clinical practice; ARCTIC INT, dual-antiplatelet treatment beyond one year after drug-eluting stent implantation; NA, not accessible; DAPT, dual antiplatelet therapy; DES-LATE, duration of clopidogrel therapy after drug-eluting stent; SECURITY, second generation drug-eluting stent implantation followed by six- versus 12-month antiplatelet therapy; ISAR-SAFE, safety and efficacy of six months dual antiplatelet therapy after drug-eluting stenting; ITALIC, is there a life for DES after discontinuation of clopidogrel; IVUS-XPL, impact of intravascular ultrasound guidance on outcomes of XIENCE PRIME stents in long lesions; OPTIDUAL, optimal dual antiplatelet therapy; NIPPON, Nobori dual antiplatelet therapy as appropriate duration.

## Supplementary Materials

The following are https://www.mdpi.com/2077-0383/9/3/652/s1, Figure S1: Assessment of publication bias using the funnel plot diagram for primary outcome.
