# Supplementary figures and images for: Efficacy and Safety of Long-Term and Short-Term Dual Antiplatelet Therapy: A Meta-Analysis of Comparison between Asians and Non-Asians
Source: J Clin Med. 2020 Feb 28;9(3):652. doi: 10.3390/jcm9030652 (PMC7141296; doi:10.3390/jcm9030652)

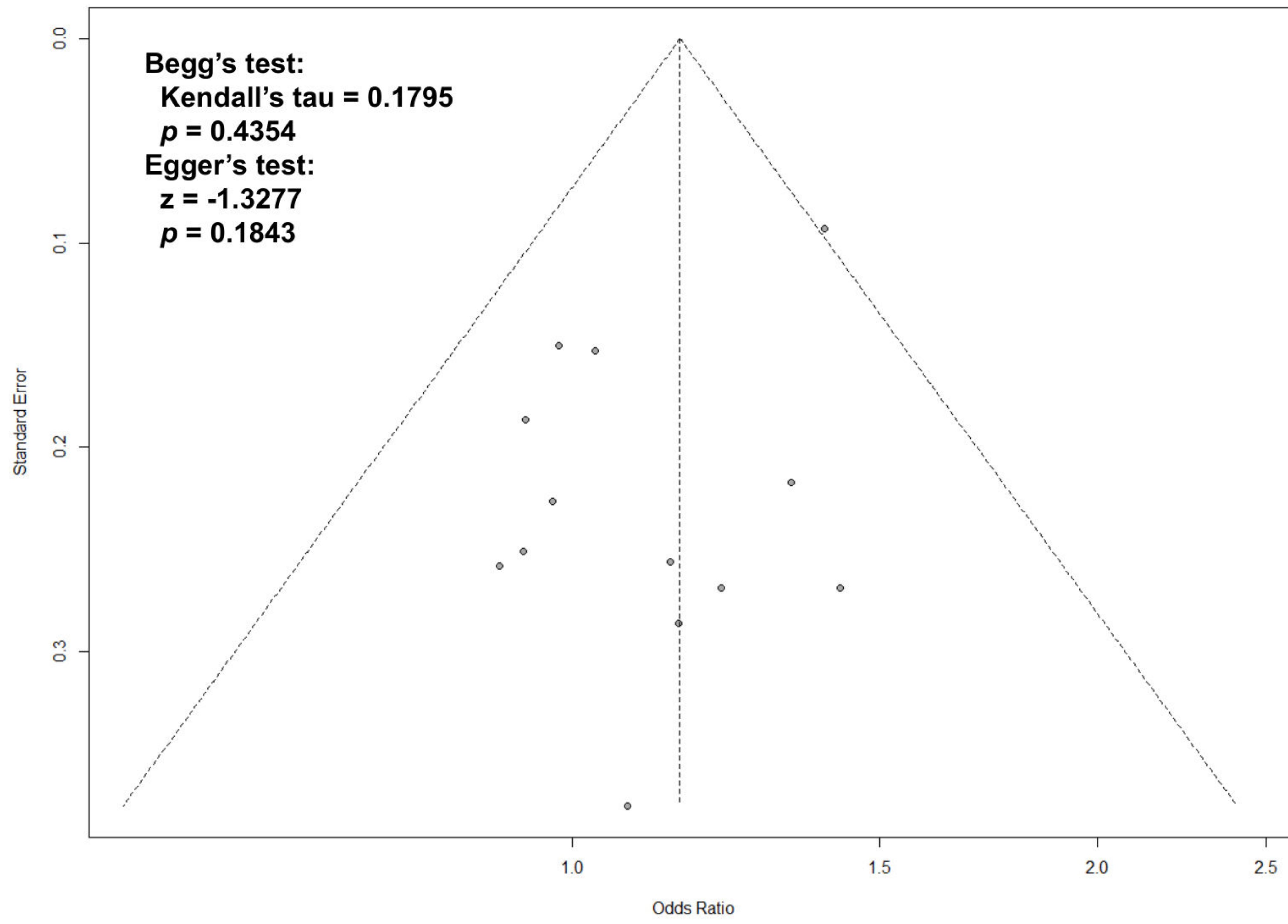

Supplement: Supplementary file 1 [file jcm-09-00652-s001.pdf]
